# Development and evaluation of a 4M taxonomy from nursing home staff text messages using a fine-tuned generative language model

**DOI:** 10.1093/jamia/ocaf006

**Published:** 2025-01-15

**Authors:** Matthew Steven Farmer, Mihail Popescu, Kimberly Powell

**Affiliations:** Sinclair School of Nursing, University of Missouri, Columbia, MO 65211, United States; Department of Biomedical Informatics, Biostatistics, and Medical Epidemiology, School of Medicine, University of Missouri, Columbia, MO 65211, United States; Sinclair School of Nursing, University of Missouri, Columbia, MO 65211, United States

**Keywords:** health services for the aged, text messaging, pattern recognition, automated, clinical decision-making, communication, ontology, taxonomy, LLM

## Abstract

**Objective:**

This study aimed to explore the utilization of a fine-tuned language model to extract expressions related to the Age-Friendly Health Systems 4M Framework (What Matters, Medication, Mentation, and Mobility) from nursing home worker text messages, deploy automated mapping of these expressions to a taxonomy, and explore the created expressions and relationships.

**Materials and Methods:**

The dataset included 21 357 text messages from healthcare workers in 12 Missouri nursing homes. A sample of 860 messages was annotated by clinical experts to form a “Gold Standard” dataset. Model performance was evaluated using classification metrics including Cohen’s Kappa (*κ*), with *κ* ≥ 0.60 as the performance threshold. The selected model was fine-tuned. Extractions were clustered, labeled, and arranged into a structured taxonomy for exploration.

**Results:**

The fine-tuned model demonstrated improved extraction of 4M content (*κ* = 0.73). Extractions were clustered and labeled, revealing large groups of expressions related to care preferences, medication adjustments, cognitive changes, and mobility issues.

**Discussion:**

The preliminary development of the 4M model and 4M taxonomy enables knowledge extraction from clinical text messages and aids future development of a 4M ontology. Results compliment themes and findings in other 4M research.

**Conclusion:**

This research underscores the need for consensus building in ontology creation and the role of language models in developing ontologies, while acknowledging their limitations in logical reasoning and ontological commitments. Further development and context expansion with expert involvement of a 4M ontology are necessary.

## Objective

Controlled Terminologies such as the Systematized Nomenclature of Medicine Clinical Terms (SNOMED CT), International Classification of Diseases (ICD), and Logical Observation Identifies Names and Codes (LOINC) assist researchers and clinicians by having a standardized representation of concepts, categories, and their relationships in specific health domains.^1^^,^^2^ This aids in data integration, reasoning, natural language processing research, and sematic interoperability. To date, there is no controlled terminology developed to describe the 4M Framework for Age-Friendly Health Systems.^3^ The 4M Framework includes four evidence-based concepts (What Matters, Medication, Mentation, and Mobility) that are fundamental to providing quality care for older adults and driving decision-making toward their health and satisfaction.^4–6^ By creating a taxonomy of expressions representing the 4M framework, researchers and clinicians can further develop analysis and interventions that promote and improve the integration of the 4M framework into clinical practice, documentation, and communications to improve care for older adults. For example, developing a standardized 4M vocabulary and describing semantic relationships between terms could lead to automated data extraction from multiple sources such as medical records, research databases, and provider notes. In addition, clinical decision support systems could leverage the taxonomy to identify gaps in documentation or clinical communication, prompting a clinician to address the gaps, potentially leading to improved outcomes.

Development of clinical ontologies is a challenging task.^7^ This is due to the complexity of the multi-disciplinary clinical domain, the need for sematic precision, consensus for standardization, and the dynamic nature of clinical science and knowledge development.^8–13^ However, recent advancements in natural language processing, specifically generative pretrained transformer (GPT) language models, may present an alternative to traditional ontology development.^14–17^ A modern ontology development can leverage these architectures in the following ways: (1) automated knowledge extraction through large scale text mining and dynamic updates to the knowledge base^18^; (2) contextual awareness and sematic understanding that removes single- or multi-word restraints on traditional natural language processing (NLP) techniques^19^; (3) scalability by adaptation in domain-specific tasks while maintaining the generalized ability to handle diverse language tasks^20^; (4) interoperability by unifying multiple ranges of medical knowledge and aspects of healthcare through the transformer model^21^; and (5) cost and resource efficiency through reducing manual labor and maintenance of complex ontology systems.^22^

These methods can depart from the traditional methods of ontology development but can be better viewed through the lens of constructivist epistemology.^7^^,^^23^ In this philosophy of science, knowledge is created and constructed by the scientific community, existing as an emergent part of human and social structures. Within the domain of medical language, taxonomies, and ontology engineering,^24^ this is an important perspective to recognize, as language itself is recognized as a human construct.^25^ The creation of any ontology, within this theory, is therefore an approximation of a reality within language which evolves alongside scientific exploration and experimentation.^26^ The utilization of a language model to support ontology learning augments this process by complementing traditional ontology development methods. The use of language models in ontology engineering is not new, but is an active research area in ontology engineering.^14^^,^^27–29^ It is currently recognized that language models show promise in automating specific tasks of ontology development but have limitations in creating consensus and persistent ontological commitments. This study focuses on developing a robust, structured taxonomy for the 4M framework that enables knowledge extraction and may facilitate the next steps of ontology creation. To achieve this, we utilize a generative language model fine-tuned on expert consensus, which then automates text extraction, labeling of hierarchical subclasses, and generation of synonyms, related words, and taxonomic relationships.

### 4M Framework and Age-Friendly Health Systems

**Figure 1.**
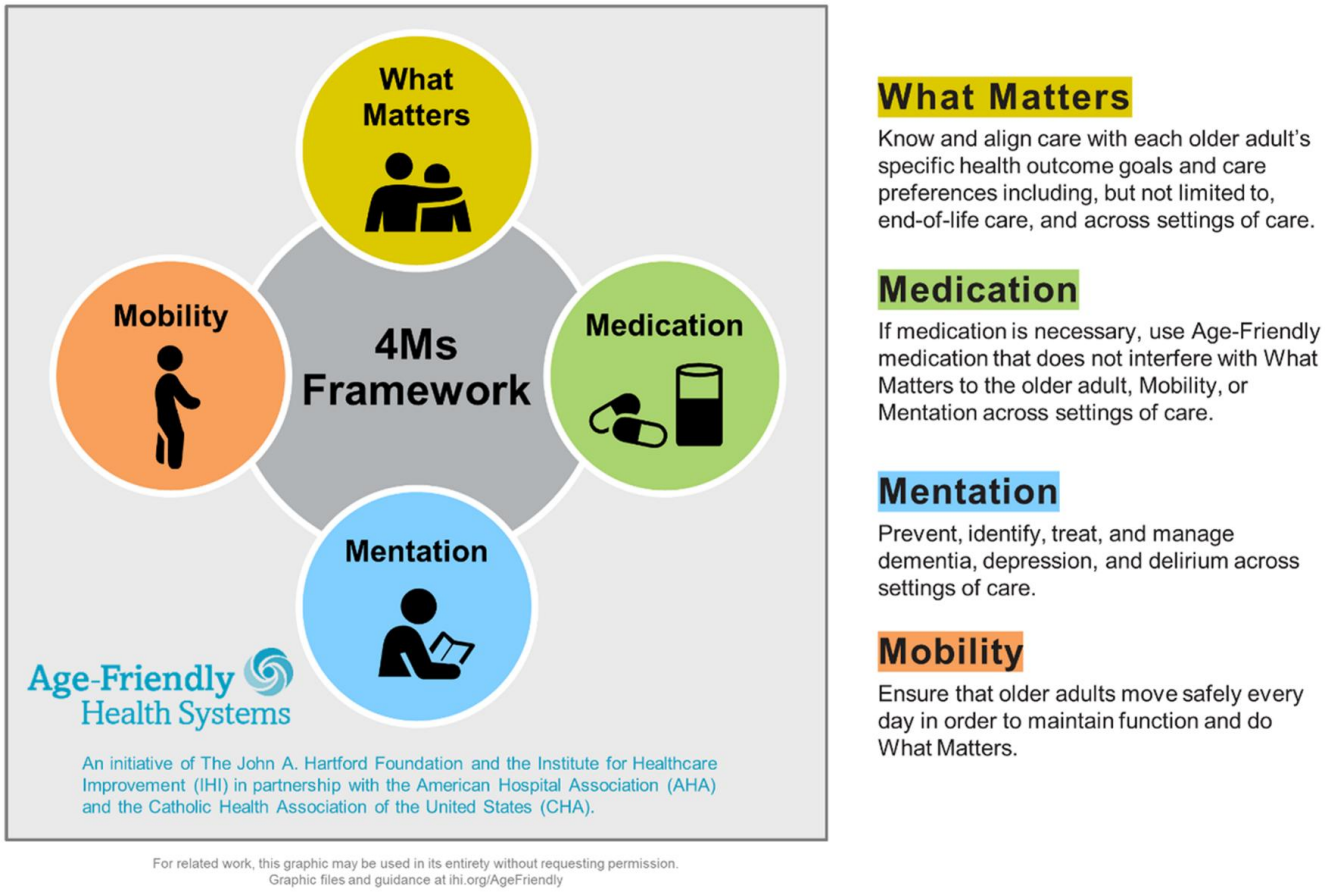
4M framework for age-friendly health systems.

The United States healthcare systems are positioned to carry an increasingly heavy volume of older adult care as our aging population grows. As the population of older adults outpaces the supply of clinicians to care for them, strategies to improve care and reduce burden on these providers must be adopted.^30^ One such strategy is the Age-friendly health systems, developed by the Institute for Healthcare Improvement (IHI) in collaboration with the John A. Hartford Foundation, which seeks to follow evidence-based practices and provide a reduced cognitive burden on clinicians when providing care for older adults.^5^ The evidence-based framework includes concepts of What Matters, Medication, Mentation, and Mobility (4Ms). In Figure 1, a visual representation from IHI is presented. These four concepts provide essential characteristics for medical decision making, clinical communication, and documentation that improve care for older adults.

### Data context and text messages

The data utilized for analysis included text messages sent by and to healthcare workers from 12 nursing homes (NH) participating in the Missouri Quality Initiative (MOQI). This initiative was a CMS-funded innovation and coordination project that began in 2012.^3^ During MOQI, one of the aspects of the improvement process was to document, through the INTERACT survey, key factors related to NH-to-hospital transfer events. Text message content was derived from a HIPAA-compliant communication tool, utilized by the workers of the participating nursing homes including features such as read receipts, response time tracking, modifying recipients, and multiuser accounts. This tool is accessed by a desktop browser or mobile device that allows nursing home workers to communicate timely resident information in a secure way. Examination of text messages is ideal in this setting due to the asynchronous nature of communication in healthcare, particularly in nursing homes where the physician or advanced practice provider may not be physically present. Furthermore, text messages offer a glimpse into the daily challenges and decision-making processes of healthcare workers, providing valuable insight not typically found in retrospective chart reviews or surveys.

### Study aims

The aims of this study was to: (1) explore the utilization of a fine-tuned small language model to extract 4M expressions from nursing home clinician text messages; (2) deploy automated mapping of these expressions to taxonomies; and (3) explore the created expressions and relationships in preparation for ontology development. Since text messages can include complex variation in communication style, typos, emotional responses, ambiguous context, short- and long-form expressions, and different work environments, a constructivist approach, leveraging generative language models is warranted to accomplish these objectives.

## Materials and methods

### Data processing and standard

The text message data were imported into a Python Jupyter notebook (version 3.12.4). The data were comprised of individual text messages and related attributes including sender, receiver, date, and timestamp. For the purposes of a larger ongoing study, these transfer events were linked to text messages sent up to two weeks prior to the transfer by the NH workers. This cutoff-time was determined by the aims of the larger study, to examine 4M communication and NH-to-hospital transfers.^3^ Preprocessing of these data and text only included removal of duplicates. To create an evaluation dataset, a sample of 860 messages, referred to as the “Gold Standard” were extracted from the full dataset and annotated by two clinical experts familiar with the 4M framework. The Gold Standard was a random sampling of 40 transfer events recorded through MOQI data and extraction of the corresponding text messages surrounding the transfer event. Currently, the 4M framework lacks a structured ontology to create specific guidelines, like words or phrases, for the annotators to extract. Therefore, the annotators reviewed the text message independently, identifying the presence of words or phrases that match any of the 4M concepts based on their understanding of the literature regarding Age-Friendly Health Systems and the 4M framework. After independent annotation, differences between the annotators were aligned via simultaneous discussion and review of the differing extractions. These words or phrases were recorded and used to calculate a total count of What Matters, Medication, Mentation, or Mobility expressions for each text message. If the message had no content related to any of the 4Ms, this was recorded as zero.

### Initial model selection and fine-tuning

Due to the identifiable nature of these data, generative language models were selected that could be ran on a local device, without access to the internet. These included small-sized, open-source licensed, generative language models that were capable of following task-specific prompts. Multiple feasibility tests were first conducted on 25% random sample of the Gold Standard dataset. Accuracy, Precision, Recall, F1, Receiver-Operator Characteristics Area-Under-Curve (ROC AUC), and Cohen’s Kappa (*κ*) were used to assess the model’s capability in extracting any 4M content that aligned with the Gold Standard. Models were evaluated with the same prompt structure and model parameters. The research team set *κ* ≥ 0.60 as the threshold to determine sufficient model performance, indicating “moderate” agreement.^31^ This threshold suggests a reliability of the extractions beyond chance that we considered acceptable in this exploratory model evaluation. Given the complexity of the 4M concepts and variability in language processing, achieving “moderate” agreement in these initial tests was deemed a reasonable benchmark, balancing the need for rigorous evaluation and practical constraints of small, locally run models. A final test was conducted against the full Gold Standard dataset to identity the best performing open-source model out of these selections.

After a final model was selected, the correct extractions and incorrect extractions (judged by the Gold Standard dataset) were labeled as “accepted” or “rejected.” This label was used to conduct parameter-efficient fine-tuning^32^ on the selected model with a reward-training methodology called Odds Ratio Preference Optimization (ORPO).^33^ The model was fine-tuned with multiple epochs then compared with the Gold Standard to evaluate changes after tuning.

Words and phrases were extracted from text messages using a structured response framework that created JSON output. Initial prompt engineering and model parameters remained unchanged to provide consistency with prior testing. This output was then assessed for presence of extraction (binary value of 4Ms concept) and then each word or phrase was counted (integer value). The binary value and integer values were used to compare against the Gold Standard dataset.

### Semi-automated taxonomy creation

After extraction, the text was processed with sentence embeddings^34^ for further language processing. First, similar extractions were removed using cosine similarity with a threshold of 0.95. Then the full dataset was split by the respective 4M classes (What Matters, Medication, Mentation, Mobility). These embeddings were then clustered with hierarchical agglomerative clustering using Euclidean distance and Ward linkage.^35^ Optimal hierarchical clustering was determined through visual review of dendrograms. Distance thresholds for four levels of hierarchy were chosen for each M allowing for discriminative separation of each cluster while avoiding excessive specific clustering. Words from each cluster were then given as a list to the language model for labeling. Each label was stored as a list to check for cosine similarity threshold of 0.9999 to avoid duplicative labeling. Labeling progressed until similarity was resolved. Prompts utilized to achieve extraction and labeling from the language model are included in the Appendix S1.

### Taxonomy exploration

With the 4Ms extracted and clustered into subclasses, we utilized the language model to define properties and relationships. Relationships were assessed by word and associated embeddings. A priori competency questions were created by the authors to determine the scope and evaluate taxonomy creation. The list of competency questions is included in the Data S1. Lastly, the final dataset was explored and visualized using descriptive statistics and network graphs.^36^^,^^37^

### Human-subjects protection

This study was approved by the University of Missouri Institutional Review Board (#2009109); PI: Kimberly Powell.

## Results

### Data processing and standard

The text message data included 21 209 text messages with dates, timestamps, text message IDs, resident IDs, and associated nursing home after duplicate removal. The Gold Standard dataset was annotated by two expert annotators independently, then differences were discussed. There were 93 differences in What Matters, 28 differences in Medication, 25 differences in Mentation, and 40 differences in Mobility. For example: annotator A extracted “symbolic disfunction” as Mentation for the text message “She is a DNR. She has DX of HTN and other symbolic dysfunction,” while annotator B did not extract this expression. Annotators agreed symbolic dysfunction should be included in Mentation. This process continued until all disagreements had resolution. The final Gold Standard dataset represented 594 expressions related to the 4M framework from the 860 text messages.

### Initial model selection and fine-tuning

Four open-source models were chosen for testing including: (1) Gemma 2 9b (9 billion parameters); (2) Gemma 2 2b; (3) Llama 3.1 8b; and (4) Mistral Nemo 12.2b.^38–40^ The model parameters included temperature = 0.0, repeat_penalty = None, top_p = 0.9, top_k = 40, seed = 418. Models extracted terms from 860 messages that matched the message IDs from the gold standard dataset. Without fine-tuning, Gemma 2 9b exhibited the best performance in 4M extraction with What Matters (*κ* = 0.41), Medication (*κ* = 0.78), Mentation (*κ* = 0.62), and Mobility (*κ* = 0.45).

Gemma 2 9b was then ORPO fine-tuned using 266 “accepted” and “rejected” examples, with 66 held for validation. Full parameters, configuration, and visualization of training loss are presented in the Appendix S1. The model was fine-tuned with 1, 2, and 4 epochs of training. Overall, 2 epochs of training demonstrated the greatest improvement in classification metrics, without overfitting. The final model resulted in What Matters (*κ* = 0.60), Medication (*κ* = 0.93), Mentation (*κ* = 0.83), and Mobility (*κ* = 0.55), with overall *κ* = 0.73. Full results of each stage are presented in Table 1.

**Table 1.** ORPO fine-tuning evaluation.^a^

		What Matters				Medication				Mentation				Mobility			
	Model	1	2	3	4	1	2	3	4	1	2	3	4	1	2	3	4
BINARY	Recall	0.42	**0.66**	0.64	**0.66**	0.97	0.94	**0.97**	**0.97**	0.67	0.75	**0.88**	**0.88**	0.35	0.42	**0.44**	**0.44**
	F1	0.50	0.63	**0.66**	**0.66**	0.82	0.90	0.94	**0.95**	0.64	0.73	**0.84**	0.79	0.48	0.57	**0.59**	0.58
	AUC	0.68	0.79	0.79	**0.80**	0.95	0.96	**0.98**	**0.98**	0.82	0.87	**0.93**	**0.93**	0.67	0.71	0.72	**0.78**
	Cohen’s Kappa	0.41	0.55	**0.60**	**0.60**	0.78	0.88	**0.93**	**0.93**	0.62	0.72	**0.83**	0.78	0.45	0.55	0.55	**0.56**
COUNT	Spearman Rho	0.44	0.57	**0.61**	**0.61**	0.82	0.89	0.93	**0.94**	0.63	0.73	**0.84**	0.79	0.50	0.60	**0.63**	0.61
	Cohen’s Kappa (binned)	0.27	0.45	0.47	**0.49**	0.53	0.75	**0.80**	**0.80**	0.50	0.62	**0.75**	0.66	0.26	0.39	**0.48**	0.43
	MAE	0.24	0.19	0.18	**0.18**	0.26	0.10	**0.09**	**0.09**	0.07	0.05	**0.03**	0.05	0.08	0.08	**0.07**	**0.07**

a Models: (1) Gemma 2 9b q4_0, (2) Gemma 2 9b ORPO q4_0 (1 epoch), (3) Gemma 2 9b ORPO q4_0 (2 epochs), (4) Gemma 2 9b ORPO q4_0 (4 epochs).

Bolded text indicates the highest score.

### Semi-automated taxonomy creation

The fine-tuned language model, hereafter referred to as the “4M Model,” was then used to extract 4M expressions from the 21 209 text messages. To contextualize the results below, summary statistics of the final dataset are as follows:

- Unique residents represented = 386
- Unique transfer events = 627 (some residents transferred multiple times)
- Unique text messages with 4M content = 7077
- Total of 4M extractions = 13 716
- Average age of resident at time of transfer = 68.16
- Count of 4M content by sender
  - Nursing Staff = 5546 (40.5%)
  - Advanced Practice Registered Nurses (APRN) = 4599 (33.6%)
  - Physician = 1276 (9.3%)
  - Other Staff (eg, Director of nursing, administrative staff, Unit manager, etc.) = 2281 (16.6%)
- Total extractions by 4M concept
  - What Matters = 2825
  - Medication = 7557
  - Mentation = 2034
  - Mobility = 1300

**Figure 2.**
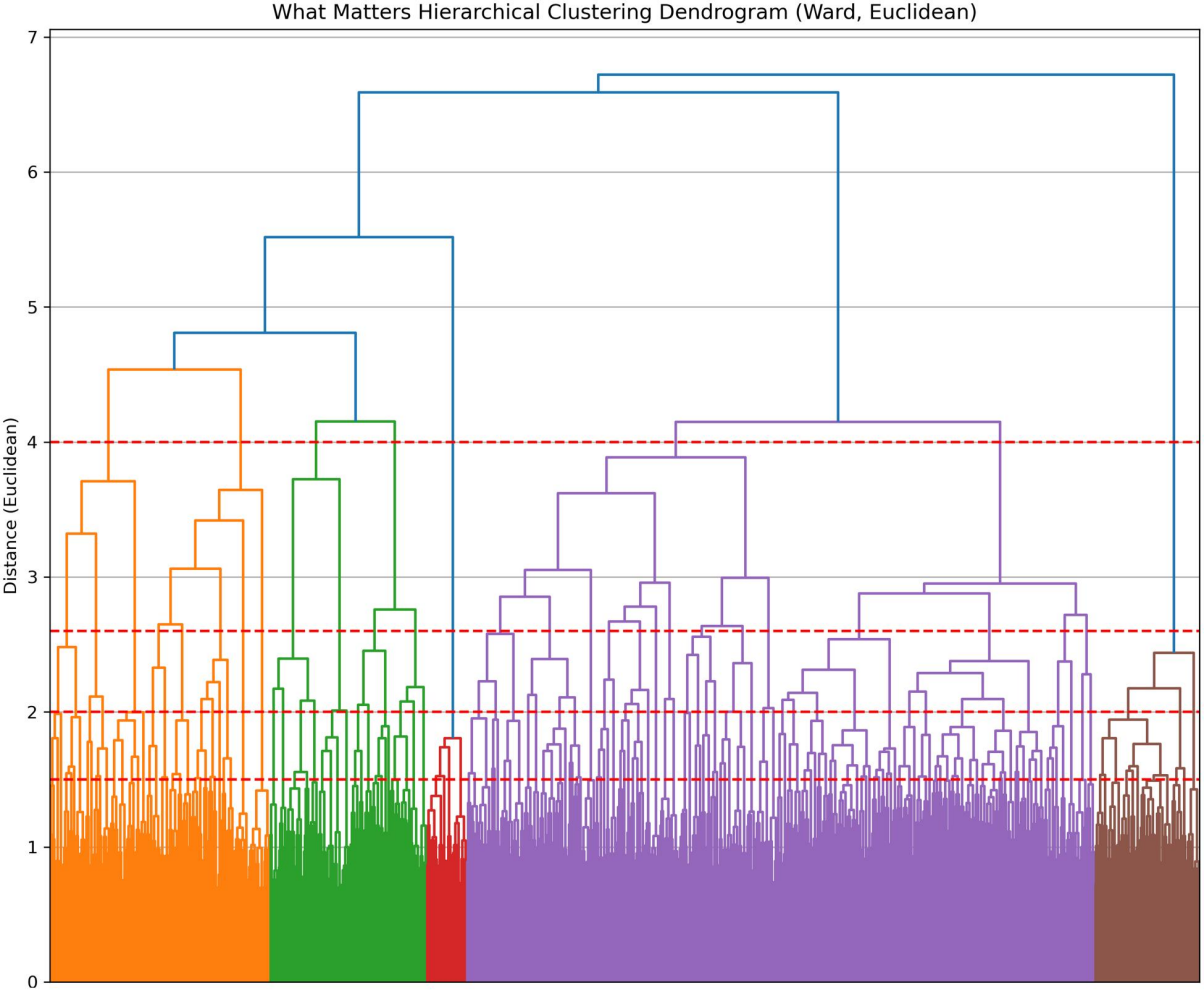
A dendrogram depicting the thresholds for hierarchical clustering.

The first level of clustering included eight clusters for What Matters, nine for Medication, four for Mentation, and four for Mobility. The unique expressions identified in the first subclass and the size of the subsequent subclasses are presented in Table 2. A dendrogram representing the thresholds for What Matters is presented in Figure 2.

**Table 2.** 4M subclass labels and distribution.

Class	Subclass Level 1	Number of Unique Expressions	*N* clusters at Level 2	*N* clusters at Level 3	*N* clusters at Level 4
What Matters	Resident Preferences and End-of-Life Care	310	28	69	176
	Resident Care Coordination	303			
	Resident Care Preferences	121			
	Resident Preferences and Family Input	102			
	Nutrition and Sleep Issues	94			
	Resident Health Status	77			
	Resident Well-being Concerns	76			
	Resident Preferences and States	39			
Medication	Medication Adjustments	729	40	69	173
	Medication Management	685			
	Pharmacological Interventions	289			
	Hypodermoclysis and Fluids Management	287			
	Resident Care Instructions	269			
	Prescription Adjustments	176			
	Scheduled Drug Administration	160			
	Potassium Management	67			
	Medication Schedules	58			
Mentation	Behavioral and Cognitive Changes	482	12	52	127
	Cognitive and Physiological Distress	146			
	Cognitive Health Status	89			
	Ocular Signs and Symptoms	28			
Mobility	Mobility Issues	261	16	40	90
	Mobility Support Needs	125			
	Patient Mobility Actions	99			
	Mobility Impairment Symptoms	65			

To illustrate the clustering and labeling, the message M19415 includes the words “pain worse with movement.” This was extracted as class = Mobility, first cluster = Mobility Impairment Symptoms, second cluster = Mobility Symptom Indicators, third cluster = Mobility Pain Indicators, fourth cluster = Mobility-Related Pain Symptoms.

### Taxonomy exploration

#### Key concepts

The 4M concept of Medication is the most frequent expression in the text message dataset including thousands of examples including prescribing, monitoring, and administration. According to the sender and receiver data, these medication messages were most frequently exchanged by nursing staff sending and advanced practice registered nurses receiving (12.1%), followed by nursing staff to DON (9.3%), and APRN to DON (4.9%). What Matters, as the second largest class, showed many expressions representing end-of-life terms, care coordination, and family preferences. For example, the level 1 cluster “Resident Preferences and End-of-Life Care” included 310 expressions. What Matters messages were primarily sent by the nursing staff to APRN roles (8.8%), followed by nursing staff to DON (7.0%) and APRN to other APRN roles (5.9%).

#### Categories

The subclasses created by the 4M model demonstrate a diversity of the clusters and the homogeny of the Medication class. The first level of clustering is shown in Table 2.

**Figure 3.**
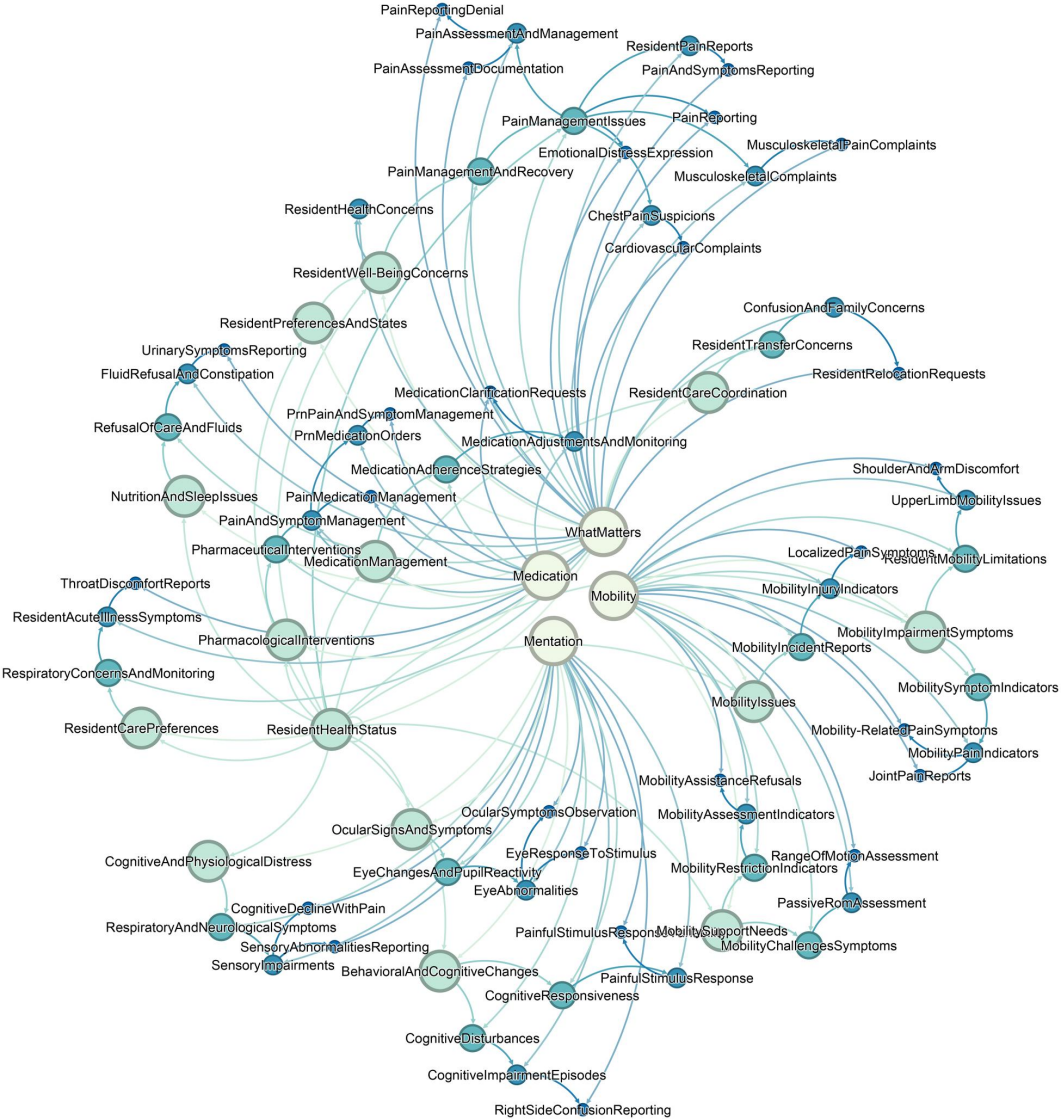
Network visualization of the 4Ms Framework components and interconnected clusters for the concept of pain.

To illustrate an exploration of this taxonomy, the word “pain” was queried for its presence in related terms or direct quotation across the data. One can assume that the concept of pain could exist in all classes. The absence of pain could be a central care preference in What Matters, driving treatment decisions for Medication, is moderated by ensuring a resident’s mental status (Mentation), and some treatment decisions may impact Mobility. The terms related to pain had the highest presence in the Medications class (*n* = 662) followed by What Matters (*n* = 494), Mobility (*n* = 113) and Mentation (*n* = 73). Figure 3 depicts how pain and related terms are found in the hierarchy of the taxonomy. This query led to a linked cluster labeled “Resident Health Status” which links the 4Ms together. Aspects of mobility challenges, pain complaints, cognitive functioning, and PRN medications are among the subclasses found in the query.

**Figure 4.**
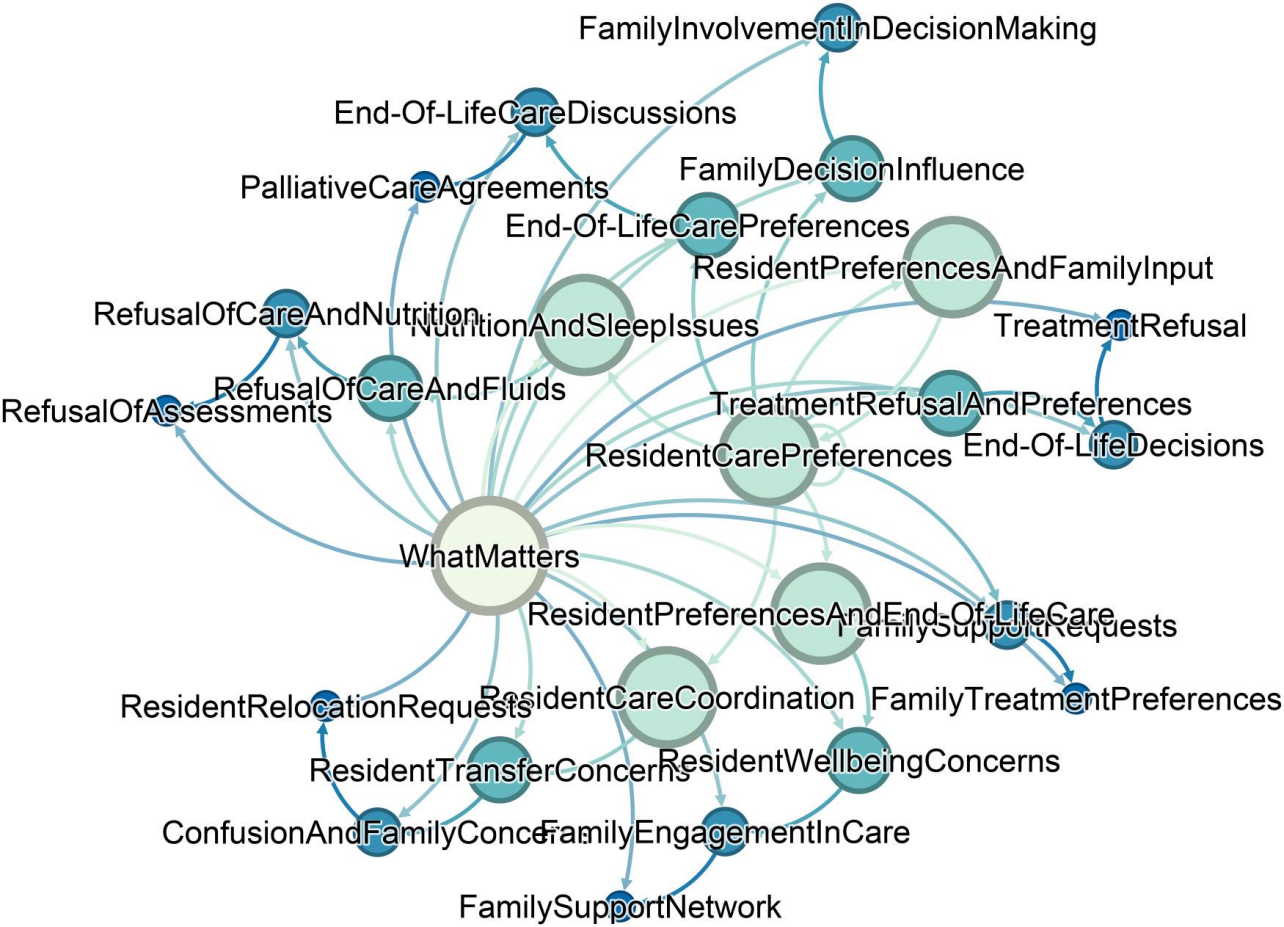
Network visualization of the 4Ms Framework components and interconnected clusters for the concept of family.

In contrast, the word “family” had multiple related expressions in What Matters and is not found in the other M’s. Seen in Figure 4, the family includes expressions related to support, decision making, end-of-life discussions, authorization, preferences, and concerns.

**Figure 5.**
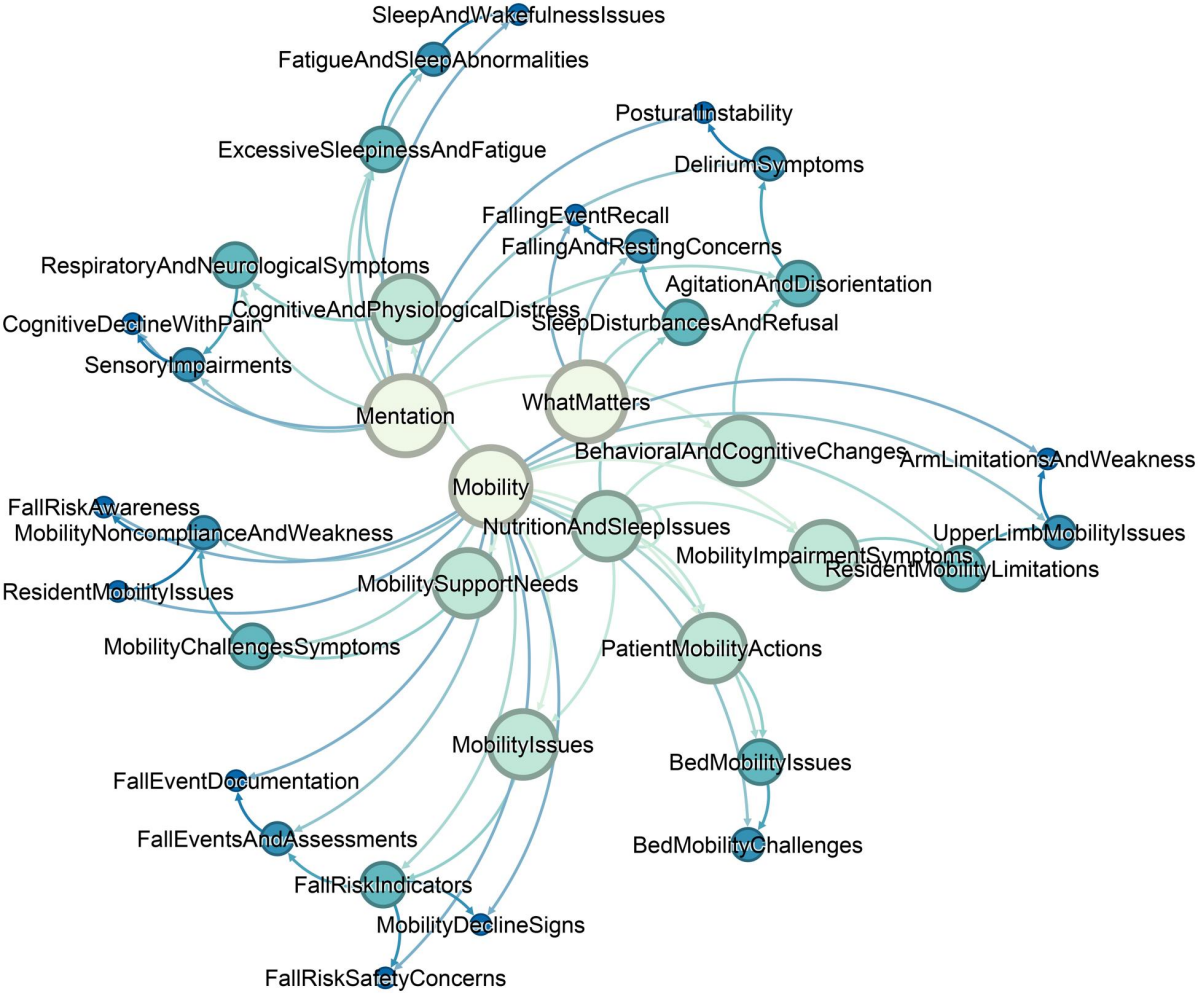
Network visualization of the 4Ms Framework components and interconnected clusters for the concept of falls.

The last example explores the term “fall.” Falls in older adults present significant complications impacting the 4M concepts. In Figure 5, we can observed the interconnectedness of falls to Mobility, What Matters, and Mentation. These concepts were linked by cognitive dysfunction, physiological changes, nutrition, and therapy adherence.

## Discussion

### Qualitative data

Current qualitative studies assessing the 4M framework reveal that many of the clusters and terms extracted from the text messages align with themes identified from residents and clinicians. For example, surveys associated with text messages utilized in this study show subthemes of family and resident preferences within What Matters which aligns with the clusters “Resident Preferences and End-of-Life Care,” “Resident Preferences and Family Input,” and “Resident Care Preferences” identified within our taxonomy.^41^ In their 2023 qualitative study, Yi et al. assembled a focus group from a purposive sample of clinicians to better understand barriers and unmet needs of inpatient care for older adults in Korea within the context of the Age-Friendly Health System Model and 4M framework. For What Matters, the participants expressed a variety of challenging individual care preferences that require focused, individualized care plans. Medication themes included complexity and challenges in medication review. Delerium management emerged as an important theme in the mentation domain. Finally, the most important theme identified in Mobility included awareness of reduced mobility and risk for falls.^42^ In our text message data, many of these themes align with the concepts and relationships we have explored. The breadth of What Matters expressions represent the complex individualized needs of each patient including family dynamics, pain, advanced care planning, and satisfaction of care.

Additional M’s have been proposed including Malnutrition^43^ and Multicomplexity,^44^ also known as the Geriatrics 5Ms.^45^ While these concepts were not included in the annotation, fine-tuning, extraction, or clustering phases of this study, there is an indication that these concepts are integrated into the taxonomy in some ways. In the first level clustering of What Matters, “Nutrition and Mobility” emerged as an independent subclass with 94 expressions. Multicomplexity includes many factors such as multimorbidity and polypharmacy.^24^ This concept was not explicitly addressed in our initial taxonomy development; however, the existing framework for the Medication concept provides a foundation for future elaboration on this phenomenon.

### Semi-automated ontology creation

The development of knowledge graphs, taxonomies, and ontologies aided by language models is not new.^27^^,^^46–48^ It is understood that the limitations of language models in ontology engineering include lack of logical reasoning, failure to adhere to ontological commitments, and inability to create consensus among experts.^27^ Our study presents an advanced approach to creating an ontology precursor. This approach involves fine-tuning a language model using a consensus Gold Standard dataset, as discussed by Mai, Chu, and Paulheim (2024). The inherent limitations are still present, due to the architecture of transformer-based language models, but the model was able to achieve higher agreement (*κ* = 0.73) with the Gold Standard. The Data S1 presents examples of model extractions compared to expert annotations, including failed extractions, hallucinations, and extractions missed by the annotators. We found that all of the models consistently performed well within the Medication and Mentation concepts yet performed poorly with What Matters and Mobility. The discrepancy likely arises from the model's training on formal language (eg, medication names, medical symptoms), while What Matters and Mobility uses less formal terminology (eg, care preferences, words related to extremities) requiring more complex reasoning. Interestingly, these are the same two areas which had the highest disagreement among the independent annotators.

### Limitations

One of the fundamental components of ontology creation is creating consensus in terminology and relationships associated with the ideas, concepts, and classifications present in the domain.^49^ Additional research and analysis have yet to be completed on a full 4M ontology to support this component. Highlighting the need for an ontology, we observed that expert annotators did not agree on independent annotated expressions before comparing them simultaneously. This illustrates the need for additional consensus-building to create a functional and robust 4M ontology.

The source of this taxonomy development was limited to unstructured text message data from nursing home workers in some Missouri nursing homes. This limits the context and depth of expressions that fully represent the 4M framework. The taxonomy would benefit from additional contexts such as inpatient communications, multidisciplinary experts, physicians’ notes, and resident/patient input. Also, the text message data in this study were for residents with a transfer event from the nursing homes participating in MOQI. The 4Ms extracted and represented in this model are, therefore, related to communication preceding a transfer event from nursing homes. Contexts such as routine communication, end-of-life discussions, safety events, etc. are not necessarily included. Lastly, these extractions are derived from NH worker communication which do not include messages to families or caregivers outside of the NH staff. The taxonomy presented here lacks input from residents, families, and other external stakeholders which would enrich the understanding of 4M concepts. Efforts to standardize the classifications and representations within the 4M ontology are far from complete.

## Conclusion

Informal clinician communication is unstructured, nuanced, and complex.^51–53^ The use of generative models can be a useful tool allowing ontologies to scale and adapt to new contexts.^14^^,^^15^^,^^22^^,^^54^^,^^55^ This study uses a language model fine-tuned on consensus data, annotated by clinical experts, to accelerate ontology engineering. This represents a novel approach to ontology development. In the current state of language models, an end-to-end ontology development still requires expert annotation and consensus. Language models lack a strict ontological commitments, can contain hallucinations, and demonstrate limitations in logical reasoning.^27^^,^^28^ Within this study, the development of a taxonomy is aided by the fine-tuned 4M language model, but a fully developed 4M ontology will still need input from experts in the field of Age-Friendly Health systems. The preliminary creation of the fine-tuned 4M model and initial taxonomy development aides in future research, ontology development, and application in resident care and precision medicine.^50^ Additional development of features, axioms, and expanding contexts of a 4M ontology are needed.

## Supplementary Material

ocaf006_Supplementary_Data

## Data Availability

The data underlying this article cannot be shared publicly due to the privacy of individuals (residents, family members, and healthcare workers) mentioned within the text messages. The data will be shared on reasonable request to the corresponding author.
