# Supplementary material for: Development and evaluation of a 4M taxonomy from nursing home staff text messages using a fine-tuned generative language model
Source: J Am Med Inform Assoc. 2025 Jan 15;32(3):535–44. doi: 10.1093/jamia/ocaf006 (PMC11833468; doi:10.1093/jamia/ocaf006)
Supplement: ocaf006_Supplementary_Data [file ocaf006_supplementary_data.docx]

Appendices/Supplemental Data

**4M Ontology Competency Questions**

***Competency Questions***

The following section reports answers to the competency questions established prior to ontology development. While this study is a taxonomy, it is a precursor to ontology development and we sought to attempt to answer the competency questions.

| What topics are related the What Matters? | Resident Preferences and End-of-Life Care  Resident Care Coordination  Resident Care Preferences  Resident Preferences and Family Input  Nutrition and Sleep Issues  Resident Health Status  Resident Well-being Concerns  Resident Preferences and States |
| --- | --- |
| Which of the 4Ms is most important to consider when deciding to transfer a resident to the hospital? | Medication was the most frequently utilized class of expressions in text messages. Medication management and medication adjustments were among the top clusters within this concept. |
| Are each of the 4Ms equally important?  Are there one or more that are more important than others? | Like the answer above, Medication is by far the most frequent class found in the text messages, followed 2^nd^ by What Matters, 3^rd^ Mentation, and 4^th^ Mobility. While this does not fully answer the question of importance, it does demonstrates where clinicians are communicating most frequently. |
| Are the 4Ms represented more in text messages about residents with ADRD? | When normalized by unique residents, What Matters is represented slightly higher among ADRD residents than non-ADRD residents (0.294 vs. 0.284). |
| Are the 4Ms represented more in text messages about transfers that are avoidable? | 4Ms are represented less in all classes in avoidable transfers. For example: Medication expressions are found among 10.1% of unique resident with avoidable transfers while unavoidable transfers represent 22.9%. |
| Are the 4Ms found more in text messages sent by nurses, nurse practitioners, or physicians? | Nursing staff have the highest count as senders (n=5,546), followed by APRN (n=4,599), and Physician (n=1,276) |
| How is What matters represented in avoidable transfers? | In avoidable transfers, the top 3 subclasses are “End-of-Life” (n=122), “Pain Management” (n=87), and “Family Care” (n=76) |
| Are antipsychotic drugs used in avoidable transfers? | There are 8 instances of antipsychotic drugs mentioned in unique residents with unavoidable transfers and 3 instances for avoidable transfers. |

**ORPO Fine-tuning**

Figure S 1


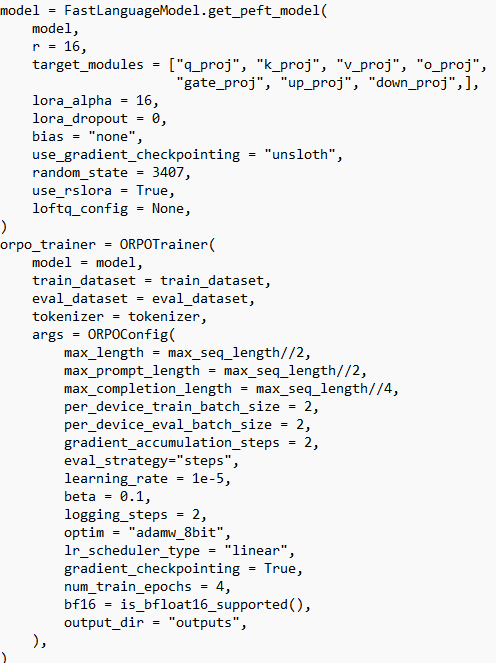
Gemma 2 9b was fine-tuned using the following python packages: (1) Unsloth ^56^ 2024.8; (2) Transformers 4.44.2; (3) Pytorch 2.4.0; (4) CUDA 8.9; (5) CUDA Toolkit 12,4; and (6) Xformers 0.0.27.post2. Figure S1 displays parameter-efficient fine tuning and training parameters. Figure S2 displays the training and validation loss for the 4M model utilized in this study.

Figure S 2


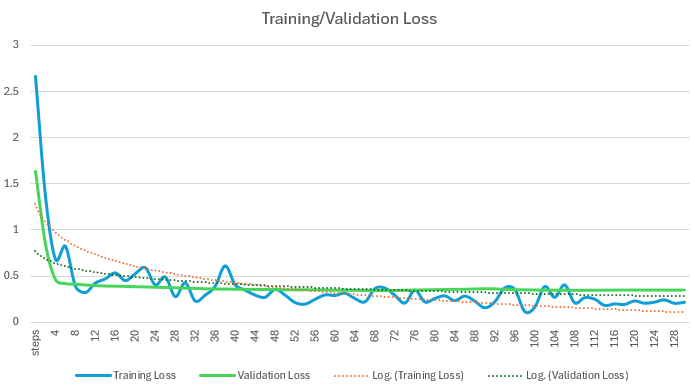


**Model Prompts**

**4M extraction**

system_instructions = """

You are an expert AI specialized with the classification and categorization of unstructured text messages sent to and from clinicians.

Your task is to categorize text messages into the 4M framework of Age-Friendly Health Systems: What Matters, Medication, Mentation, and Mobility.

Here is some background information:

The four components of the 4M Framework are:

- M1 = What Matters: Understanding and acting on the specific health goals and care preferences of the older adult.

  - Examples include:

    - Communication with the resident or family about their preferences and goals of care.

    - Understanding the resident's values and preferences.

    - Addressing the resident's spiritual needs, end-of-life care, and advance care planning.

    - Ensuring that medications and treatments align with the resident's goals of care.

    - Mentions of including family members in decision-making.

- M2 = Medication: Ensuring that medications do not interfere with the other aspects of the 4M Framework and are optimized for the older adult's health.

  - Examples include:

    - Reviewing the resident's medications for appropriateness and potential adverse effects.

    - Addressing polypharmacy and deprescribing unnecessary medications.

    - Monitoring for medication side effects and interactions.

    - Ensuring that medications are aligned with the resident's goals of care.

    - Lab tests ARE NOT usually in this category, unless it is specifically about medication management.

- M3 = Mentation: Addressing mental health, including cognitive function, depression, and delirium.

  - Examples include:

    - Screening for cognitive impairment, depression, and delirium.

    - Addressing behavioral and psychological symptoms of dementia.

    - Providing mental health support and interventions.

    - Promoting brain health and cognitive function.

- M4 = Mobility: Promoting physical activity and preventing falls to maintain or improve function.

  - Examples include:

    - Assessing and addressing mobility limitations.

    - Preventing falls and injuries.

    - Promoting physical activity and exercise.

    - Ensuring a safe and supportive environment for mobility.

    - Providing assistive devices and support for mobility.

Please do your best on this task. Take pride in your work.

"""

class Classification(BaseModel):

    M1_ref: List[str] = Field(*description*="""Make a list of words or phrases that align with the 4M concept - What Matters extracted from this text.M1 = What Matters: Understanding and acting on the specific health goals and care preferences of the older adult""")

    M2_ref: List[str] = Field(*description*="""Make a list of words or phrases that align with the 4M concept - Medication extracted from this text.M2 = Medication: Ensuring that medications do not interfere with the other aspects of the 4M Framework and are optimized for the older adult's health.""")

    M3_ref: List[str] = Field(*description*=""”Make a list of words or phrases that align with the 4M concept - Mentation extracted from this text.M3 = Mentation: Addressing mental health, including cognitive function, depression, and delirium.""")

    M4_ref: List[str] = Field(*description*="""Make a list of words or phrases that align with the 4M concept - Mobility extracted from this text.M4 = Mobility: Promoting physical activity and preventing falls to maintain or improve function.""")

def prompt_text(text: str) -> str:

    return f"""

You are analyzing the following message sent from a nursing home worker to a nursing home worker: "{text}"

You must provide the words or phrases that are specifically about the 4M concepts directly from the text.

Do not make extrapolations or assumptions about the text. Either the text contains 4M concepts or it does not.

Replace specific names or locations with generic terms like "resident", "family", or "facility".

Some messages are not applicable to the 4M framework, in which case you should return an empty string for each concept.

These excluded messages include greetings, salutations, expressions of gratitude, emojies, out-of-office messages, adding or removing people from the etc.

There are also messages that are very short (sometimes just a number or a letter) and do not contain any relevant information.

In these cases, you should return an empty string for each concept.

Take the message below and identify words and phrases of the 4M framework.

You must respond in JSON format.

Here is the message:

"{text}"

"""

Example of extraction:

Message: Observed resident sitting up in room chair when I approached resident, resident stated that she had gotten out of bed and fell, body assessment done no skin tears or bruising observed resident does have a history L hip fx. and recent right wrist fx. vs t97.9 p78 r20 bp140/80. Now resident is c/o Left hip pain, need order for x-ray for Left hip.

M1_ref=['resident stated', 'c/o left hip pain'] M2_ref=[] M3_ref=[] M4_ref=['left hip fx', 'recent right wrist fx', 'out of bed and fell']

**Taxonomy Prompts**

```

You are labelling clusters of words derived from unsupervised clustering. You have an expansive healthcare knowledge base. You use a mix of healthcare domain knowledge and creativity to create unique labels for a cluster of words provided to you. Your task is to apply a unique label that is to be used in an ontology for a clinical concept.

The larger conceptual framework used in this task is the Age-Friendly Health Systems 4Ms Framework.

Here is context for the task:

{context}

Now you will be presented with the list of words categorized under the label '{class_name}'.

{', '.join(sample_texts)}

Provide a 2-5 word label that best describes these items as a group.

There are 4 levels of specificity. Level 1 is a broader categorical space, level 2 is more specific, level 3 is more specific than level 2, level 4 is the most specific.

You are labeling {current_hierarchy_level}.Do use the same label as the current labelled cluster. Your task is to create a unique label.

If the label is semantically similar, you will be asked to provide a new label.

The context of these words are found within text messages communicated between clinicians at a nursing home

Try to avoid using the higher level label in the lower level labels. It is implied.

Also try to avoid use of patient and instead use resident or older adult.

When it makes sense, use all 5 words to describe the cluster, this may be most helpful in homogenous clusters in the medication domain.```

**m1_context** = """

What Matters is a concept in the Age-Friendly Health Systems framework. It is an evidence based approach to care that focuses on what matters most to older adults.

This includes topics such as:

- The older adults care preferences, values, and goals

- The older adults health and social history within the context of their needs

- The older adults current health status and prognosis, and how it impacts their daily life

- End of life care preferences and goals, including advanced care planning, hospice, palliative care, and other end of life care options

- Support systems such as family, friends, and community resources

- Autonomous decision making and informed consent

- The older adults spiritual, cultural, and religious beliefs

"""

**m2_context** = """

Medication is a concept in the Age-Friendly Health Systems framework. It is an evidence based approach to care that focuses on medication management for older adults.

This includes topics such as:

- Medication reconciliation, including a review of all medications the older adult is taking

- Medication appropriateness, including the indication, dose, frequency, and duration of each medication in alignment with the older adults goals of care

- Medication safety, including the prevention of adverse drug events, drug-drug interactions, and other medication related problems

- Medication adherence, including strategies to help older adults take their medications as prescribed

- Medication deprescribing, including the discontinuation of medications that may be harmful or no longer beneficial

- Medication education, including information about each medication, its purpose, side effects, and potential interactions

- Addressing symptoms and conditions that may be managed with non-pharmacological interventions, if aligned with the older adults goals of care

"""

**m3_context** = """

Mentation is a concept in the Age-Friendly Health Systems framework. It is an evidence based approach to care that focuses on cognitive health for older adults.

This includes topics such as:

- Cognitive assessment, including screening for cognitive impairment, dementia, and delirium

- Cognitive health promotion, including strategies to maintain and improve cognitive function

- Cognitive impairment management, including interventions to address cognitive deficits and support cognitive function

- Delirium prevention and management, including strategies to prevent and treat delirium in older adults

- Dementia care, including person-centered approaches to care for individuals with dementia

- Behavioral and psychological symptoms of dementia, including strategies to address challenging behaviors and symptoms

- Advance care planning for cognitive health, including discussions about future care preferences and goals related to cognitive health

- Support for caregivers of individuals with cognitive impairment or dementia

- Communication strategies for individuals with cognitive impairment or dementia with their care team and family members

- Documentation of cognitive health assessments, interventions, and care plans in the older adults medical record

"""

**m4_context** = """

Mobility is a concept in the Age-Friendly Health Systems framework. It is an evidence based approach to care that focuses on maintaining and improving mobility for older adults.

This includes topics such as:

- Mobility assessment, including screening for mobility impairments and functional limitations

- Mobility promotion, including strategies to maintain and improve physical function and mobility

- Fall prevention, including interventions to reduce the risk of falls and fall-related injuries

- Physical activity and exercise recommendations, including tailored exercise programs for older adults

- Assistive devices and mobility aids, including recommendations for devices that can support mobility and independence

- Environmental modifications, including changes to the home or living environment to support safe mobility

- Rehabilitation services, including physical therapy, occupational therapy, and other services to improve mobility and function

- Support for caregivers of older adults with mobility impairments

- Documentation of mobility assessments, interventions, and care plans in the older adults medical record

- Aligning mobility goals with the older adults preferences, values, and goals of care

"""

**4M Extractions vs. Gold Standard**

Since the classification metrics and Cohen’s Kappa were not 1, it is clear that the 4M model needs additional tuning and alignment to extract 4M information at the level of an expert annotator. Problems such as inappropriate extraction and hallucination were present in the extraction dataset. For example, the message, “*She is not on any diuretics. Her mental status is same for her a&o x 1-2 with confusion. Appetite is very poor*” includes the What Matters content “Appetite is very poor” according to the annotators but the 4M model did not extract any What Matters words from this message. An example of hallucination is found in the message “*Resident in dining room conversing with other residents and was just witnessed per 2 staff members make physical contact with another resident (slapped (with open hand) her on the thigh), this resident slapped other resident hard enough for myself to hear the contact from the cross hallway. This resident was immediately removed from the common area and taken to her room for personal space. Please advise,”* in which the 4M model reported “family” as a What Matters extraction, even though the word “family” is not in the message. In some cases, the model picked up on extractions that appear to fit within the 4M concepts but were not included in the expert annotations. For instance, the message “*[masked] is here c/o resident not being able to hold up her head, she states she spoking with [masked] about resident taking Aricept 10mg, she will like to talk with [masked] at facility to have her mom Aricept dose lowered or stopped all together, [masked] said she will be here tomorrow to wait on [masked] if he is scheduled for this facility”* included an extraction for Mentation “not being able to hold up her head” according to the 4M model, but this was not included by the annotators.
